# Serum α-chlorofatty acid as a biomarker for baseline subclinical cardiovascular disease in systemic lupus erythematosus

**DOI:** 10.1186/ar3956

**Published:** 2012-09-27

**Authors:** MA Mahieu, C Guild, CJ Albert, G Kondos, J Carr, D Edmundowicz, DA Ford, R Ramsey-Goldman

**Affiliations:** 1 Northwestern University Feinberg School of Medicine, Chicago, IL, USA; 2 Saint Louis University, Saint Louis, MO, USA; 3 University of Illinois Chicago, IL, USA; 4 Temple University School of Medicine, Philadelphia, PA, USA

## Objective

α-chlorofatty acid (α-ClFA) is one product of myeloperoxidase activity *in vivo *during atherogenesis [1]. Our study investigates whether serum α-ClFA may be a biomarker for subclinical cardiovascular disease (CVD) in patients with systemic lupus erythematosus (SLE).

## Methods

One hundred and eighty-five women with SLE and 186 controls participated in this ancillary study of the Study of Lupus Vascular and Bone Long-term Endpoints (SOLVABLE). Data collection included demographic information, CVD and SLE risk factors, and baseline laboratory assessments. α-ClFA was measured in stored serum by liquid chromatography-electrospray ionization mass spectrometry with selected reaction monitoring detections. Each sample was run in triplicate. Coronary artery calcium (CAC) and aorta calcium (AC) were measured by electron beam computed tomography or multi-detector computed tomography. Calcium scores were calculated using the Agatston method. Outcome measures were the presence of higher risk CAC or AC scores (CAC >10 or AC >100) versus lower risk scores (CAC ≤10 or AC ≤100) [2]. Significant associations were identified with descriptive characteristics, univariate, and multivariate analyses.

## Results

Cases had higher baseline levels of α-ClFA than controls (42.2 ± 19.2 fmol/μl vs. 34.5 ± 10.9 fmol/μl, *P *= 0.014). Cases with lower risk CAC and AC scores had statistically higher levels of α-ClFA compared with controls, while cases and controls with higher risk CAC and AC scores had similar α-ClFA levels (Table 1). In multivariate analyses, SLE had the strongest independent association with higher risk CAC scores, followed by dyslipidemia and age (Table 2). SLE also had the strongest association with higher risk AC scores, followed by history of tobacco use, age, and C-reactive protein level (Table 3). α-ClFA was not independently associated with higher risk CAC or AC scores.

**Table 1:** Baseline serum α-ClFA levels (fmol/μl) in cases and controls by higher risk versus lower risk CAC and AC scores

Calcium score	Cases	Controls	*P *value
CAC >10	43.3 ± 21.5	44.0 ± 14.8	0.951
CAC ≤10	42.0 ± 17.6	33.7 ± 10.5	0.010
AC >100	39.3 ± 7.8	37.6 ± 13.1	0.743
AC ≤100	40.4 ± 12.3	33.9 ± 10.5	0.023

**Table 2:** Multivariate analysis for higher risk CAC scores

Variable	OR	95% CI
SLE	5.81	2.28 to 14.83
Dyslipidemia^a^	5.67	1.50 to 21.36
Age	1.11	1.05 to 1.17
α-ClFA	1.00	0.99 to 1.01

^a^Dyslipidemia defined as total cholesterol >200, low-density lipoprotein >100, high-density lipoprotein <40, triglyceride >150, or lipid-lowering medication use.

**Table 3:** Multivariate analysis for higher risk AC scores

Variable	OR	95% CI
SLE	3.73	1.59 to 8.78
Tobacco use	2.31	1.13 to 4.74
Age	1.17	1.10 to 1.25
C-reactive protein	1.05	1.01 to 1.11
α-ClFA	1.01	0.99 to 1.02

## Conclusion

SLE had the strongest independent association with the presence of higher risk subclinical CVD, while serum α-ClFA levels were not independently associated at baseline.
